# Computed tomography findings and preoperative risk factors for mortality of total anomalous pulmonary venous connection

**DOI:** 10.1007/s10554-018-1405-2

**Published:** 2018-06-25

**Authors:** Yonghua Xiang, Guanxun Cheng, Ke Jin, Xuehua Zhang, Yuan Yang

**Affiliations:** 1 Department of Radiology, Nanfang Hospital, Southern Medical University, Guangzhou, China; 2 Department of Radiology, Hunan Children’s Hospital, University of South China, Changsha, China; 3 Department of Ultrasound, Hunan Children’s Hospital, University of South China, Changsha, China; 4 Department of Health Statistics, The Second Xiangya Hospital of Central South University, Changsha, China

**Keywords:** Total anomalous pulmonary venous connection, Follow-up study, Mortality, Heart defects, Congenital

## Abstract

Detailed preoperative imaging of total anomalous pulmonary venous connection (TAPVC) is critical to ensuring adequate surgical planning and preoperative decision making. The purpose of this study was to describe the computed tomography findings of TAPVC and identify morphologic death risk factors. We conducted a retrospective study included 70 patients with TAPVC between May 2014 and June 2017 in Hunan Children’s Hospital. All available clinical data and computed tomography imaging were reviewed, and survival time was followed-up. Life Tables analysis was used to estimate survival rates. Patient survival was described with Kaplan–Meier curves. Cox Regression model was used to test the potential risk factors. TAPVC was subdivided into four types. Of 70 cases, 42 (60%) had supracardiac, 13 (18.6%) had cardiac, 8 (11.4%) had infracardiac, and 7 (10%) had mixed type. Pulmonary venous obstruction (PVO) was found in 30 (42.9%) of 70 patients in this group. Of all concurrent abnormalities, atrial septal defect (ASD) was the most common (98.6%), followed by patent ductus arteriosus (PDA; 31, 44.3%), and persistent left superior vena cava (PLSVC; 5, 7.1%). 1, 3, 6 and 12-month survival rates were 76, 61, 49, and 38% respectively. Risk factors for mortality in multivariable analysis comprised PVO, McGoon index (MGI), and mode of delivery. Various concurrent abnormalities and great morphological heterogeneity were observed in patients with TAPVC. Patients with TAPVC had a highest mortality in the neonatal period. PVO, smaller MGI and caesarean are important predictors for mortality.

## Introduction

Total anomalous pulmonary venous connection (TAPVC) is a rare malformation of congenital heart disease in which all pulmonary veins connect directly to the systemic venous circulation, and not the left atrium [1]. It account for approximately 1–3% of congenital heart diseases [2]. According to the drainage site of the pulmonary veins, it was classified into supracardiac, cardiac, infracardiac, and mixed types [3]. There was great morphological heterogeneity of the pulmonary venous connections, which has been implicated as the causative factor of the high mortality [4, 5]. Echocardiography is regarded as the preferred diagnostic modality in patients with TAPVC, but it is a challenge in complete depiction of some complex feature of TAPVC [6]. Computed tomography angiography (CTA) is then an accurate, noninvasive, economical diagnostic modality in preoperative evaluation of TAPVC [7, 8]. It helps to improve diagnostic accuracy and have significant advantages in accurately evaluating the courses of pulmonary venous connections [9, 10]. An understanding of complete anatomical details and physiological assessment are critical to medical and surgical management [5].The purpose of this study was to describe the CTA findings of TAPVC and identify morphologic death risk factors.

## Patients and methods

### Patients

This study was approved by the Ethics Committee of Hunan Children’s Hospital with a waiver of informed consent. From May 2014 to June 2017, 70 patients with TAPVC were identified in Hunan Children’s Hospital. The patients included 47 male and 23 female, aged from 1 day to 5 years, median age was 23 days. Patients with TAPVC who were confirmed by surgery or were identified simultaneously by CTA and Ultrasound were included in the study. Cases with functionally univentricular circulation, heterotaxy syndrome, common atrium or pulmonary atresia were excluded when testing potential risk factors, because these additional lesions have a highly significant influence on overall outcome [11].

### Data collection

The clinical records including admission age, gender, birth weight, and mode of delivery were collected from the hospital database. Radiological findings were reviewed and recorded by two radiologists with more than 10 years of experience in cardiac imaging. When the opinions were not unified, they reached a consensus through consultation. The common abnormal signs of TAPVC were selected as the observation indicators. Finally, seven CT indicators and three clinical indicators were selected for analysis. Measuring the inner diameters of common pulmonary arteries (PA), right PA (RPA), left PA (LPA) and ascending aorta (AA) at aorta-pulmonary artery level, descending aorta (DA) at the level of the diaphragm, the right (RD) and left ventricular inner diameter (LD) of middle cavity on four-chamber view, the thickness of right ventricular wall (RT) and left (LT), and then to calculate the ratio of common PA to AA (PA/AA), RD to LD (RD/LD) and RT to LT (RT/LT). McGoon index (MGI) was calculated as the sum of RPA and LPA divided by DA. The data including PA/AA, RD/LD, RT/LT and MGI measured by the two radiologists were averaged. The ratios were preferred to minimize the effect of age when testing potential risk factors. PVO was considered if the drainage veins were reduced by 50% or more from the largest measured dimension. Drainage veins connecting to portal vein were also taken as an indicator of PVO, because obstruction results from the high resistance of portal venous flow needing to traverse through the liver parenchyma before returning to the heart [12]. Mild hypoplasia of the individual pulmonary vein wasn’t included in PVO in this study.

### Follow-up

Forty of the 70 patients underwent surgical repair. Their cut-off point of survival time was the day before operation. Calculating survival time from birth to the day before operation served as censored cases. Of the remaining 30 patients, we all followed-up by telephone. One patient was lost to follow-up, and calculating survival time from birth to admission date served as censored case. The other 29 patients all died within 6.5 months of birth, and calculating survival time from birth to death served as events have occurred. Censored cases were less than 60%.

### Statistical analysis

Data were collected and analyzed with SPSS18. Normally distributed continuous variables were described as mean ± SD. Student *t* tests were used to compare the differences between groups. For skewed continuous variables, median was used to describe distributions. Descriptive statistics for categorical variables were reported as frequency/percentage and were compared by use of the Pearson X^2^ or Fisher exact test. Life Tables analysis was used to estimate survival rate. Patient survival was described with Kaplan–Meier curves. Cox Regression model was used to test the potential risk factors. Variables for the multivariable analysis were chosen if *P* < 0.05 on univariable analysis and there were < 5% missing data. Values of *P* < 0.05 were considered statistically significant.

## Results

The clinical characteristics, concurrent abnormalities and calculated data of all TAPVC patients were shown in Table 1. Various concurrent abnormalities were observed in patients with TAPVC. Of all concurrent abnormalities, ASD is the most common (69, 98.6%), followed by PDA (31, 44.3%), and PLSVC (5, 7.1%).

**Table 1:** The clinical characteristics, concurrent abnormalities and calculated data of all 70 patients with TAPVC

Covariate	Total	Covariate	Total
Age at admission		Coexistent anomalies	
Range	1 day–5 years	ASD (PFO)	69 (98.6%)
Median	23 (days)	PDA	31 (44.3%)
Gender, n (%)		PLSVC	5 (7.1%)
Female	23 (32.9%)	CoA	3 (4.3%)
Male	47 (67.1%)	PVS	3 (4.3%)
Birth weight (kg)		PA	2 (2.9%)
Mean (SD)	3.19 (0.37)	SV	2 (2.9%)
Low birth weight (n)	4	Dextrocardia	2 (2.9%)
Delivery mode, n (%)		VSD	2 (2.9%)
Natural labor	43 (61.4%)	ARSA	2 (2.9%)
Caesarean/(preterm)	26 (37.2%)/1	Heterotaxy	2 (2.9%)
Unknown	1 (1.4%)	ECD	1 (1.4%)
PA/AA (mean (SD))	1.62 (0.27)	CA	1 (1.4%)
MGI (mean (SD))	2.07 (0.52)	UCS	1 (1.4%)
RD/LD (mean (SD))	1.60 (0.36)	DORV	1 (1.4%)
RT/LT (mean (SD))	1.47 (0.39)		

*PA*/*AA* the ratio of pulmonary artery/ascending aorta; MGI, (LPA + RPA)/DA; *RD*/*LD* the ratio of inner diameter of right ventricle/left, *RT*/*LT* the ratio of right ventricular wall thickness/left, *ASD* atrial septal defect, *PFO* patent foramen ovale, *PDA* patent ductus arteriosus, *PLSVC* persistent left superior vena cava, *CoA* coarctation of aorta, *PVS* pulmonary valve stenosis, *PA* pulmonary atresia, *SV* single ventricle, *VSD* ventricular septal defect, *ARSA* aberrant right subclavian artery, *DORV* double outlet right ventricle, *ECD* endocardial cushions defect, *CA* common atrium, *UCS* unroofed coronary sinus

### Pulmonary venous connection and PVO

TAPVC was subdivided into four types according to the drainage site of the pulmonary veins. The detailed site of drainage and position of PVO were depicted in Table 2. Of 70 cases, 42 (60%) had supracardiac, 13 (18.6%) had cardiac, 8 (11.4%) had infracardiac, and 7 (10%) had mixed connections. In supracardiac TAPVC, the confluent pulmonary vein (CPV) directly connected to the innominate vein (IV; n = 37), the superior vena cava (SVC; n = 2) via an ascending vertical vein (VV). The pulmonary vein (PV) respectively drained into IV and SVC (n = 2), IV and azygous vein (AV; n = 1). In cardiac TAPVC, the CPV drained into the coronary sinus (CS) in ten patients. In the remaining three patients, individual PVs were observed to drain directly into the right atrium (RA). In infracardiac TAPVC, the CPV drained into a descending VV through the diaphragm. The drainage of the VV can be categorized into 4 types: connected to the portal vein (n = 3), connected to the hepatic vein (HV; n = 3), connected to the ductus venosus (n = 1), and connected to the inferior vena cava (IVC; n = 1). In mixed TAPVC, the drainage vein respectively connected to CS and IV (n = 5), SVC and RA (n = 1), SVC and portal vein and hepatic vein (n = 1).

**Table 2:** The pulmonary venous connection and the position of PVO

Type	N (%)	Site of drainage (n)	PVO (%)	Position of PVO (n)
Supracardiac	42 (60%)	IV (37)	22 (52.4%)	VV at LPA (15)
		SVC (2)		VV at LPA to IV (5)
		IV + SVC (2)		VV at RPA (1)
		IV + AV (1)		IV to SVC (1)
Intracardiac	13 (18.6%)	CS (10)	0 (0%)	
		RA (3)		
Infracardiac	8 (11.4%)	PV (3)	7 (87.5%)	Connect to PV (3)
		HV (3)		VV to HV (3)
		IVC (1)		Lower VV (1)
		DV (1)		
Mixed	7 (10%)	CS + IV (5)	1 (14.3%)	Connect to PV (1)
		SVC + RA (1)		
		SVC + PV + HV (1)		

*IV* innominate vein, *SVC* superior vena cava, *AV* azygous vein, *CS* coronary sinus, *RA* right atrium, *PV* portal vein, *HV* hepatic vein, *IVC* inferior vena cava, *DV* ductus venosus, *VV* verital vein, *LPA* left pulmonary artery, *RPA* right pulmonary artery

PVO was found in 30 (42.9%) of 70 patients in this group. The PVO occurred in 22 (52.4%) of 42 supracardiac cases, 0 of 13 cardiac cases, 7 (87.5%) of 8 infracardiac cases, and 1 (14.3%) of 7 mixed cases. There was significant difference in the presence of PVO among the four types (P < 0.001). In supracardiac type, PVO occurred in the VV at its intersection with the LPA (n = 15), the far part of the VV between LPA and IV (n = 5), the VV at its intersection with the RPA (n = 1), and the IV (n = 1). In infracardiac type, PVO occurred in the orifice of the VV to HV (n = 3), and resulted from the VV connects to the portal vein (n = 4). Seven patients complicated with mild dysplasia of a single PV, five of them complicated with PVO at the same time, and the other two cases weren’t included in PVO in this study.

### Estimate of survival and risk factors associated with mortality

**Fig. 1:**
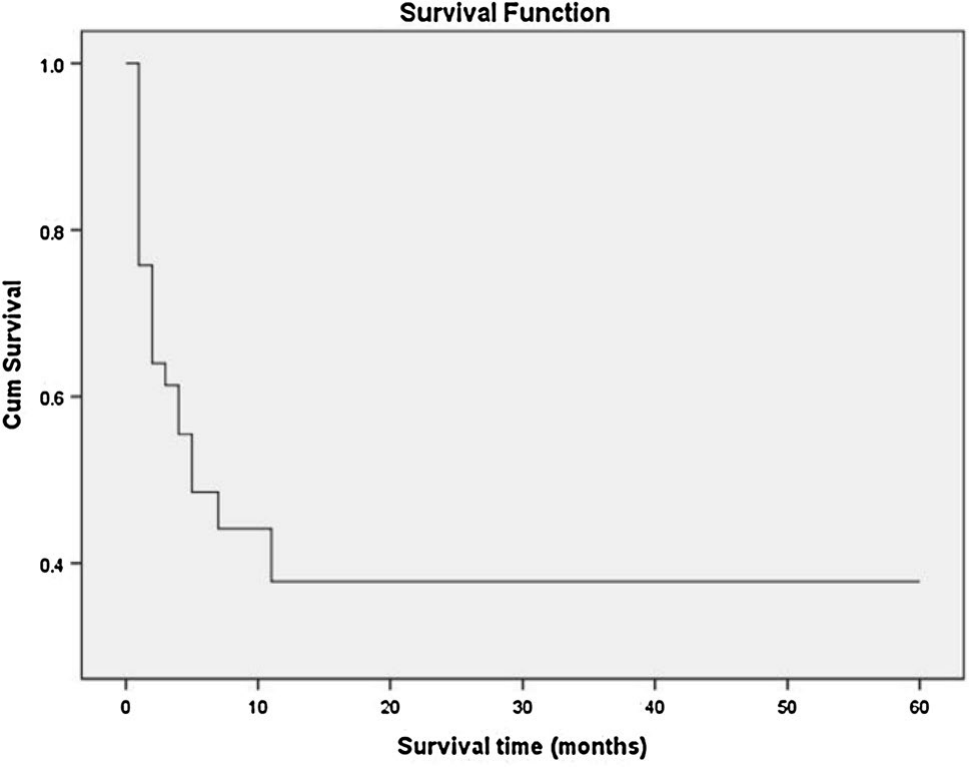
Life Tables analysis was used to estimate survival rate. Survival Function of patients with TAPVC. There is a steep early hazard for death in the neonatal period. The mortality tends to be stable after 12 months old

Seventy patients were used to estimate survival rate. Estimates of 1-, 3-, 6-months, and 1-year survival were 76, 61, 49, and 38% respectively (Fig. 1). The median survival time was 4.8 months. Patients with TAPVC had a higher mortality in the neonatal period. More than half of the patients died within 6 months. The mortality tends to be stable after 1 year old.

**Fig. 2:**
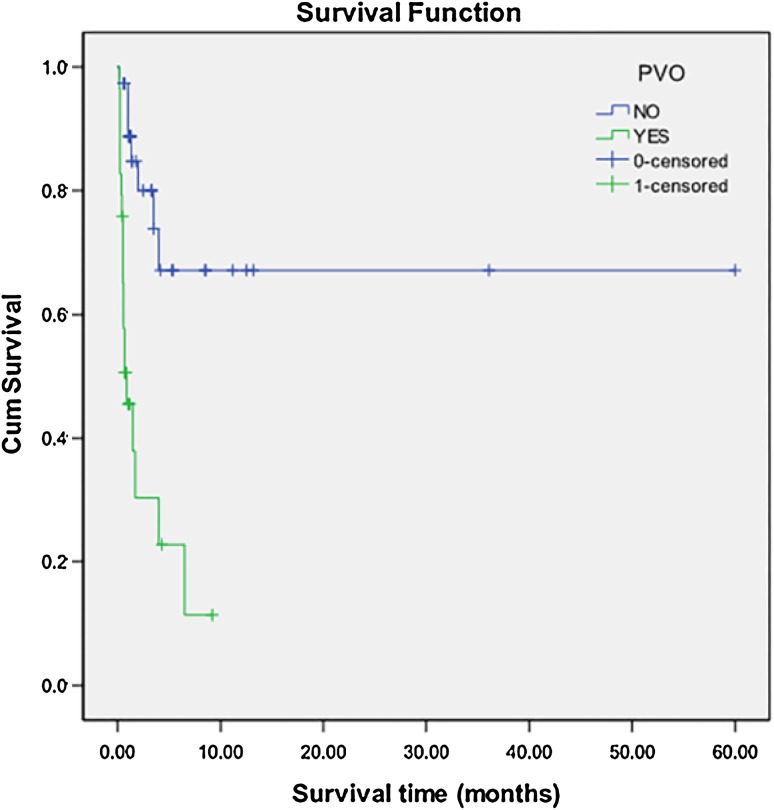
Kaplan–Meier analysis of overall survival between the patients with PVO and those without PVO. The overall survival in patients with PVO is lower than those without PVO. (Log Rank X^2^ = 23.344, *P* = 0.000)

**Fig. 3:**
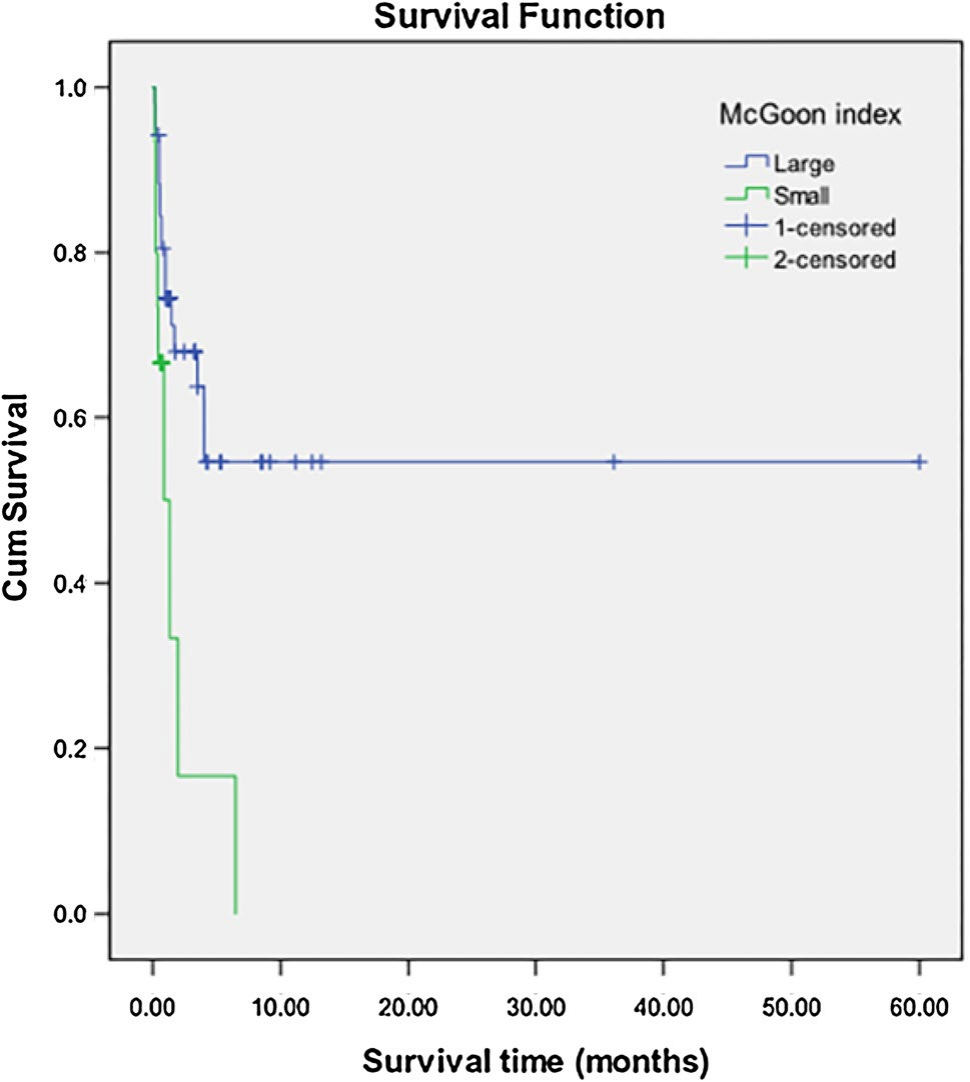
Kaplan–Meier analysis of overall survival between the patients with small MGI and those with large MGI. The overall survival in patients with small MGI is lower than those with large MGI. (Log Rank X^2^ = 8.492, *P* = 0.004)

**Fig. 4:**
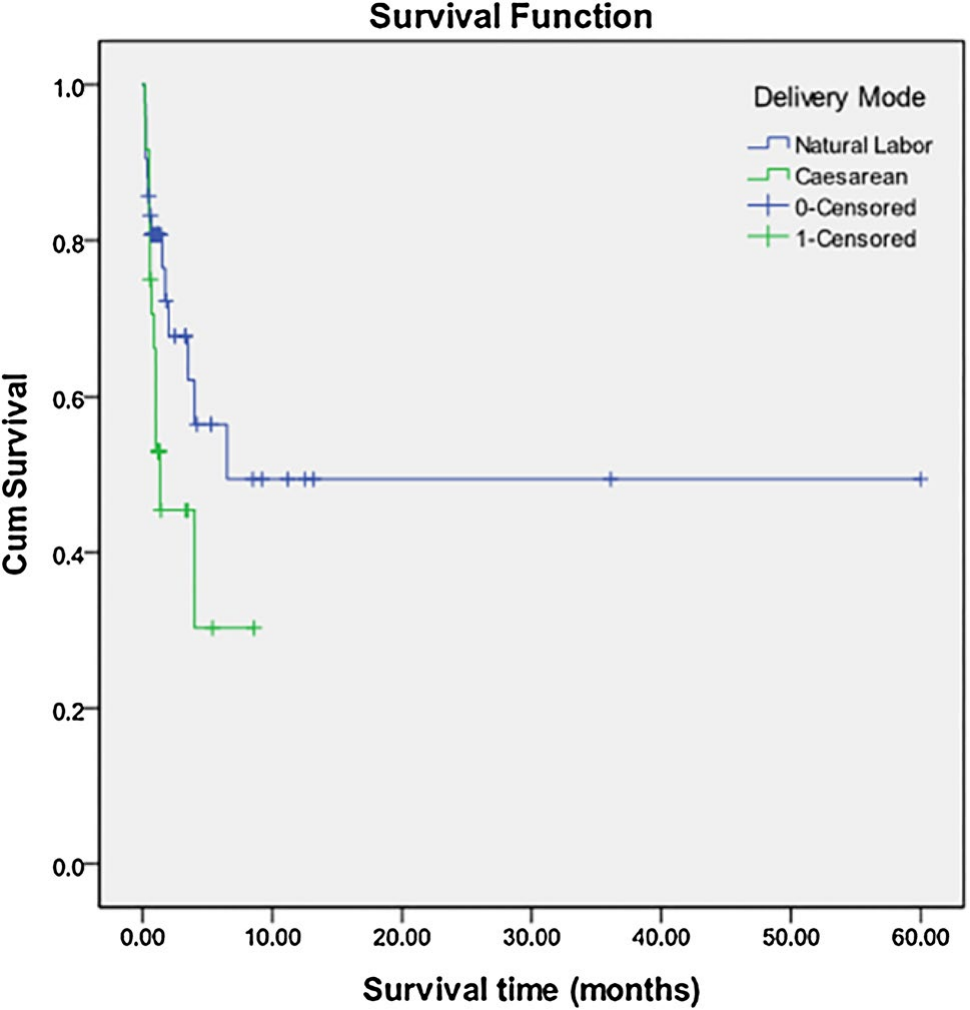
Kaplan–Meier analysis of overall survival between the patients by caesarean and those by natural labor. The overall survival in patients by caesarean is lower than those by natural labor. (Log Rank X^2^ = 4.213, *P* = 0.040)

Three patients with common atrium, single ventricle, heterotaxy syndrome or pulmonary atresia were excluded, and 67 cases with TAPVC were included to analyze risk factors for death. Twenty-six (38.8%) of the 67 patients had died. Of one patient was lost to follow-up. Risk factors for death by univariable and multivariable analysis were shown in Table 3. Patients with PVO, smaller MGI, and caesarean had a higher likelihood of death (Figs. 2, 3, 4).

**Table 3:** Risk factors associated with mortality in the 67 cases (3 cases were excluded) with TAPVC

Variables	Univariable analysis			Multivariable analysis		
	HR	95% CI	P value	HR	95% CI	P value
PVO	5.602	2.417–12.986	0.000	4.122	1.560-10.889	0.004
MGI	3.157	1.386–7.195	0.006	3.294	1.139–9.521	0.028
Delivery mode	2.144	1.009–4.557	0.047	3.385	1.467–7.814	0.004
Infracardiac	3.231	1.149–9.087	0.026	2.554	0.791–8.243	0.117
Pulmonary lesions	1.138	0.510–2.539	0.751			
Gender	0.476	0.194–1.166	0.104			
Birth weight	1.576	0.564–4.405	0.386			
PA/AA	0.487	0.168–1.414	0.186			
RD/LD	1.334	0.460–3.869	0.595			
RT/LT	1.114	0.469–2.646	0.807			

*HR* hazard ratio, *CI* confidence interval

## Discussion

### Risk factors for death

In this study, we found that PVO, smaller MGI, caesarean, and infracardiac type were significantly associated with an increased risk of death in the univariable analysis, but infracardiac type was not an independent risk factor for death in the multivariable analysis. The presence of PVO is very important in the physiopathology of TAPVC, and it cause elevated pressure in the pulmonary venous territory, elevated pressure in the pulmonary capillary bed, pulmonary edema, pulmonary hypertension, and right heart failure with leftward shift of the interventricular septum and low systemic output. PVO was found to be associated with an increased risk of death in our study, which was consistent with previous reports [11, 13]. We found that infracardiac type was not an independent risk factor for death in the multivariable analysis. The impact of connection type on outcomes has been debated [14, 15], partly because results are confounded by the multicollinearity between PVO and certain connection types that often precludes discrimination of both as potential risk factors within a multivariable model. Infracardiac type is far more likely to have connections to the portal system and longer anomalous venous channels, which are prone to developing PVO. PVO occurred in 87.5% of infracardiac cases in this study. So we speculated that both PVO and infracardiac type had been neutralized as potential risk factors in the multivariable analysis.

MGI has been used as a morphologic indicator in evaluating the development status of pulmonary artery in patients with congenital heart disease. The development status of pulmonary artery is one of the most important criteria for decision-making strategy and predicting postoperative outcome in congenital heart disease. The MGI was also useful for assessing pulmonary vascular remodeling in fetuses with congenital diaphragmatic hernia [16]. Patients with TAPVC frequently have impaired pulmonary vascular development. We found that smaller MGI was associated with an increased risk of death in this study. The previous study suggested that the development of pulmonary arteries and pulmonary veins correlated with each other, and excellent correlations between the size of pulmonary veins and pulmonary arteries were found [17]. Bando et al. [18] indicated that diffuse pulmonary venous narrowing was a risk factor for death. Jenkins et al. [19] found that individual PV size was an important predictor of survival in neonates with TAPVC. To our knowledge, this is the first time that smaller MGI was described as a risk factor for death of patients with TAPVC.

We found that mode of delivery is an independent risk factor for death of patients with TAPVC. The risk for death of caesarean was higher than natural labor. Could the risk for death be attributed to caesarean events per se? Or did the fetus with TAPVC per se bring about caesarean? Unfortunately, we didn’t collect the detailed information about the caesarean, and didn’t understand the reasons performing caesarean. The previous report [20] indicated that neonatal respiratory morbidity is increased generally in the group of elective cesarean delivery patients, which resulted in increased neonatal intensive care unit admission. Caesarean is the independent risk factors which caused neonatal respiratory tract infection [21]. The incidence of persistent pulmonary hypertension of the newborn was almost fivefold higher among neonatal delivered by elective cesarean than those delivered vaginally [22]. Neonatal mortality rates were higher among infants delivered by cesarean section than for those delivered vaginally, when death due to congenital malformations and events with Apgar scores less than four were excluded [23]. In our study, 3 of 4 patients with low birth weight were birth by caesarean, so the fetus with TAPVC per se maybe attribute to caesarean, but there is no difference of average birth weight between the group of caesarean and natural labor. We found relatively earlier admission age and lighter average admission weight in the group of caesarean. We speculate that caesarean was likely to exacerbate the symptoms of patients with TAPVC, and then the patients should be treated earlier. Any way the precise mechanisms remain unclear, and it is worthy of further discussion.

### Survival rate

In our study, estimates of 1-, 3-, 6-months, and 1-year survival were 76, 61, 49, and 38% respectively. The median survival time was 4.8 months. There is a steep early hazard for death in the neonatal period. More than half of the patients died within 6 months. But there is still a survival rate of 38% without operative intervention after 1 year, and since then the survival rate tends to be stable. Historically, patients with TAPVC have a high mortality rate of approximately 80% in the first year of life without intervention [24].

Our study showed a lower mortality rate in the first year of life than previously reported. This may be related to advance in prenatal diagnosis, attending, and medical stabilization. None of patients with cardiac type died, but 75% of patients with infracardiac type died, and there was a similar mortality between supracardiac and mixed type before operation in our group. We also found that the patients with older age at admission had larger ASD. The previous report showed patients with supracardiac and cardiac type can live for older age than those with infracardiac and mixed type [25]. The most important factors affecting the survival period are large ASD and normal to near normal pulmonary artery pressure [26]. Several cases of adult untreated TAPVC have been reported [26, 27], even a few cases were diagnosed after 60 years of age due to large ASD.

### Morphology

This study showed great morphological heterogeneity and provided a detailed describe of how frequently each subtype occurs. In our group, 60% of patients are supracardiac, 18.6% are cardiac, 11.4% are infracardiac, and 10% are mixed type. The supracardiac type of connection is the most common, followed by the cardiac, infracardiac, and the mixed type, which is consistent with previous reports [10, 13, 28]. TAPVC can occur in conjunction with a wide variety of cardiac abnormalities. ASD is the most common concurrent abnormality, and all our patients present ASD except a case with common atrium. PDA has also a high incidence of 44.3% in this study, and the patients with PDA have a smaller ASD than those without PDA. A right-to-left shunt is obligatory for survival, and it usually occurs at atrial level through an ASD, complete absence of the atrial septum, or less commonly a PDA [6]. The presence of PDA may relate to pulmonary pressure. We speculated remaining open of the ductus arteriosus can alleviate the pulmonary hypertension and provide more blood flow for systemic circulation.

### TAPVC with PVO

In our study, PVO has a total incidence of 42.9%, and infracardiac type has the highest incidence of PVO, up to 87.5%, followed by 52.4% of supracardiac type and 14.3% of mixed type, but none of the patients with cardiac type presents PVO. PVO was found in 25–50% of TAPVC patients [13, 29, 30], and dramatically alters the presentation, physiology, and outcomes. PVO is far more likely to occur in patients with infracardiac TAPVC and presents in up to 85% of infracardiac cases [6, 11]. Generally, the longer of the drainage vein connecting to the RA, the higher incidence of PVO. The infracardiac type has the longest drainage vein of all types. If the drainage vein connects to the portal vein, obstruction results from the high resistance of portal venous flow needing to traverse through the liver parenchyma before returning to the heart [12]. Our study showed the position of PVO remains relatively constant. In supracardiac TAPVC, PVO frequently located in the VV (95.5%), especially located in the VV at its intersection with the LPA (68.2%) due to the compress of the LPA. In infracardiac cases, PVO often occurred at the distal orifice of the descending VV, or resulted from the VV connect to the portal vein.

### Study limitations

This is a single-center retrospective study. inevitably, some cases with TAPVC would have been missed. Cases used to estimate survival rate were nonrandomized. That could affect the outcome of survival rate. TAPVC has complex clinical manifestations and morphology, and many factors can affect its survival. Limited clinical and CT variables were selected for Cox Regression analysis due to a relatively small sample size. Some of the selected variables might affect each other in a Cox Regression mode. TAPVC often combined with various malformations. Although the cases combined with malformations of high risk for death were excluded, the other concurrent abnormalities could still affect survival outcome.

## Conclusions

Various concurrent abnormalities and great morphological heterogeneity were observed in patients with TAPVC. CTA has an important role in providing accurate anatomical details and morphological evaluation, and it is helpful for surgical planning. Patients with TAPVC have a steep early hazard for death in the neonatal period. Morphological features including PVO and smaller MGI are important predictors for mortality. Caesarean is an independent risk factor for death, but the precise mechanisms remain unclear, it requires further study.
